# Epigenetic age acceleration and methylation differences in IgG4-related cholangitis and primary sclerosing cholangitis

**DOI:** 10.1186/s13148-024-01803-x

**Published:** 2025-01-16

**Authors:** Alexandra Noble, Rodrigo Motta, Silvia Cabras, Belen Moron Flores, Jan Nowak, Aleksandra Glapa-Nowak, Alessandra Geremia, Jack Satsangi, Emma Culver

**Affiliations:** 1 Translational Gastroenterology and Liver Unit, John Radcliffe Hospital, Headley Way, Headington, Oxford, OX3 9DU UK; 2 Nuffield Department of Medicine, University of Oxford, Oxford, UK; 3 Department of Pediatric Gastroenterology and Metabolic Diseases, Poznan University of Medical Sciences, Poznan, Poland

**Keywords:** PSC, IgG4-RD, DNA methylation, Infinium Methylation EPIC arrays, HLA, Epigenetic clock, Age acceleration

## Abstract

**Background:**

IgG4-related cholangitis (IgG4-SC) and primary sclerosing cholangitis (PSC) are chronic fibro-inflammatory hepatobiliary conditions, with genetic, environmental, and immunologic risk factors, in which epigenetic alterations may provide insights into pathophysiology and novel biomarkers. This study is the first to assess methylation signatures in IgG4-SC.

**Results:**

Whole blood DNA methylation profiling and genotyping was performed in 264 individuals; 47 with IgG4-SC, 65 with PSC, 64 with ulcerative colitis (UC), and 88 healthy controls. We identified 19 significant methylation differences between IgG4-SC and controls and 38 between PSC and controls. IgG4-SC and PSC shared 8 probes. Inflammatory genes (including *CEP97*, *IFNAR1*, *TXK*, *HERC6*, *C5orf36*, *PYY*, and *MTRNR2L1*) were predominantly involved in dysregulated methylation. Epigenetic age acceleration was observed in patients with IgG4-SC, but not in those with PSC or UC. meQTL analyses to identify genetic determinants of methylation revealed a strong human leucocyte antigen (HLA) signal in both PSC and IgG4-SC (*HLA-DQB2*, *HLA-DPA1*, *HLA-F* and *HLA-DRA*).

**Conclusions:**

We identify novel epigenetic alterations in IgG4-SC and PSC, with biological age acceleration in IgG4-SC, providing insights into disease pathogenesis, and highlight the role of genetic variation especially within the HLA region in shaping the methylome.

**Supplementary Information:**

The online version contains supplementary material available at 10.1186/s13148-024-01803-x.

## Background

Chronic inflammation plays a central role in the pathogenesis of numerous diseases, contributing to an elevated risk of age-related conditions, such as cardiovascular disorders and cancer [1, 2], as well as end-organ dysfunction and failure [3, 4]. IgG4-related disease (IgG4-RD) and primary sclerosing cholangitis (PSC) are chronic immune-mediated conditions characterised by a complex interplay of genetic and environmental factors. These diseases are associated with significant morbidity and mortality, including an increased risk of malignancies and organ failure [5, 6]. Both diseases show a male predominance [7, 8], and are marked by abnormalities in the immune system, including enhanced autoreactivity and changes in B and CD4 + T cells [9–11]. Furthermore, HLA complex genes are associated with disease susceptibility [12–14]. Overall, there are three risk loci associated with IgG4-RD [14, 15] while PSC has more than 20 risk loci, but they account for less than 10% of disease liability [12]. Similarly, genetic variants represent approximately 20% of disease susceptibility to inflammatory bowel disease (IBD) [16].

In addition to genetic predisposition, environmental factors are likely involved in disease pathogenesis and may act through epigenetic mechanisms, such as in primary biliary cholangitis [17, 18]. DNA methylation, histone modifications and expression of microRNAs represent key regulatory processes influenced by external and internal stimuli. Several studies have identified a consistent and replicable methylome in IBD, with changes being associated with response to treatment, and the value of epigenetic alterations as diagnostic and prognostic biomarkers is emerging [19–21]. A notable example is the differentiation of PSC and concomitant ulcerative colitis (UC) from those with UC alone based on, DNA methylation patterns in the *NINJ2* gene [22]. These findings show the importance of epigenetic alterations not only in disease pathophysiology, but also as possible diagnostic markers.

Nevertheless, the methylomes of PSC and IgG4-related sclerosing cholangitis (IgG4-SC), one of its main differential diagnoses, remain underexplored. Here, we investigate DNA methylation patterns in PSC and IgG4-SC using peripheral blood samples and comparing them to patients with UC and healthy controls (HC) with the aims to elucidate the underlying pathogenesis, identify novel disease biomarkers and uncover potential therapeutic targets.

## Methods

### Recruitment of patients and controls

Patients with IgG4-SC, PSC and UC as well as HC were recruited from outpatient clinics at the John Radcliffe Hospital, Oxford, UK. The IgG4-SC group had 47 patients, the PSC group had 65 patients, the UC group had 64 patients, and there were 88 healthy controls. The colitis extent and activity in the PSC, and UC patients were well matched. Sample sizes were based on power calculations using data from previous work by this group [19, 20].

### Diagnostic criteria

Patients with IgG4-SC received a diagnosis based on the HISORt criteria [23] for IgG4-related pancreatic and biliary disease [24]. Organ damage and response to treatment were assessed using the IgG4-responder index (IgG4-RI) [25]. Disease activity was also analysed among patients with IgG4-RD, and it was defined as an IgG4-RI score above 3 points. PSC patients were diagnosed in accordance with the EASL guidelines on sclerosing cholangitis [26] and UC patients were diagnosed according to consensus guidelines [27]. Colitis extent was assessed during the last colonoscopy and disease activity by the Partial Mayo Score at the time of blood collection. HCs had no known immune or inflammatory disease.

### DNA extraction

DNA was extracted from whole blood using the Qiagen Puregene Blood core Kit C (Qiagen) and bisulfite converted using EZ-96 DNA methylation kits (Zymo Research).

### Genotyping

Genotyping was performed with the Global Screening Array-24 v3.0 with multi-disease drop in panel (Illumina) with initial processing in Genome Studio v2.0.4, and subsequent processing and analysis in PLINK [28] v1.07. Single nucleotide polymorphisms (SNPs) with minor allele frequencies < 5% or missing in > 2% of samples were excluded, as were samples with a sex mismatch, missing > 2% of SNP calls, or where ethnicity was estimated as non-European using data from the 1000 Genomes project (phase 3) [29].

All analyses were performed in R v4.3.1 (R Core Team, Vienna, Austria), and statistical significance is defined using the Holm method unless stated otherwise.

### Methylation

Genome-wide analysis of DNA methylation was conducted on DNA extracted from peripheral blood, assayed with Infinium Methylation EPIC arrays (Illumina) with samples randomised with respect to diagnosis. Minfi [30] was used to read raw data, perform standard quality control and functional normalisation. ComBat [31] was used to correct batch effects for array, slide, and processing batch. Deconvolution of proportions of granulocytes, B-cells, CD4 and CD8 T cells, monocytes, and natural killer cells was performed with the Houseman method [32], using the FlowSorted.Blood.EPIC package [33].

Epigenome-wide associations were tested using limma [34], by linear models with Empirical Bayes correction, including age, sex and the first principal component of deconvoluted cellular proportions as covariates. Statistical genome-wide significance was determined using the Holm method. Genomic inflation was measured using quantile–quantile plots.

GO terms analysis was performed with Goseq [35], using the number of probes per gene as a bias weighting factor, the top 5000 differentially methylated CpG sites between each disease groups were used for analysis with significance was determined by FDR < 0.05. The package DMRcate [36] was used to compute differentially methylated regions (DMRs), significance was determined by FDR < 0.005.

### Methylation quantitative trait loci

Within each cohort, methylation data was subsetted (nominal *p* < 0.01) following epigenome-wide analysis (subseted methylation sites *n* = 19418 IgG4-SC and *n* = 28834 PSC). Methylation quantitative trait loci (meQTLs) were analysed by linear regression. Each methylation probe was tested against each SNP within 150 kb, with methylation regressed on genotype with age, sex, and the first principal component of deconvoluted cellular proportions as covariates.

### Epigenetic ageing

Methylation age was predicted with 334 available probes from the 353 in Horvath’s epigenetic clock [37] using wateRmelon [38]. A linear regression with chronological age was used to produce the expected methylation age, and age acceleration was defined as observed methylation age minus the expected methylation age.

## Results

### Demographics

Demographics and relevant clinical findings for each group follow the known epidemiological distributions for these diseases. The IgG4-SC group had a median age at diagnosis of 63 years (IQR 12), with 81% being male, 30% were taking immunosuppressive drugs and 53% were in disease remission at the time of blood donation for this study. The PSC group had a median age at diagnosis of 46 years (IQR 36), 68% were male and 26% used immunosuppressants. Patients with UC had a median age at diagnosis of 24 years (IQR 15), 48% were male and 42% used immunosuppressants. (Supplementary Table 1). Patients with PSC-UC and UC were well matched and had a similar distribution and activity of colonic involvement; and no patient had been listed for liver transplant or had transplantation (data not shown).

### Cell type proportions differences between IgG4-SC, PSC and UC

Cell type proportions were estimated for each of the disease groups, differences were mostly observed between IgG4-SC and healthy control. Specific cell types included CD4T (*p* = 0.00004), CD8T (*p* = 0.0001) and neutrophils (*p* = 0.00004) (Supplementary Fig. 1).

### Epigenome-wide associations found in IgG4-SC

**Fig. 1:**
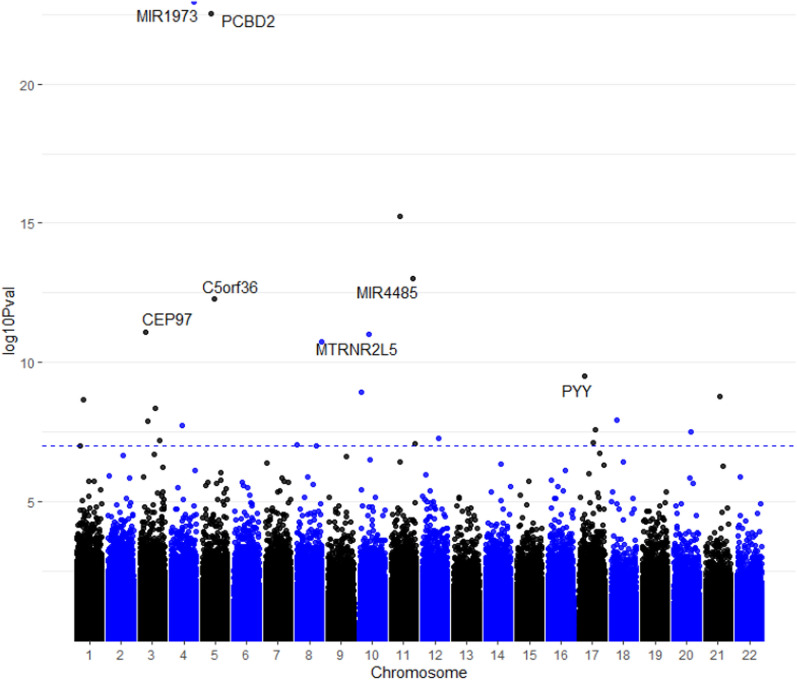
EWAS Manhattan plot of differentially methylated probes in patients with IgG4-SC compared to healthy controls

Comparison between patients with IgG4-SC and HC showed 19 DMPs in the first group after Holm correction (Table 1, Fig. 1, Supplementary Fig. 2B). Among the most significant CpG sites, the main genes identified were *MIR1973* (*p* = 9.5 × 10^−18^), *PCBD2* (*p* = 2.58 × 10^−17^), *MIR1974/C5orf36* (*p* = 4.69 × 10^−7^) and *CEP97* (*p* = 6.83 × 10^−6^). A total of three differentially DMR’s were observed in the IgG4-SC group, these included *MTRNR2L8*, *MTRNR2L13* and *CEP97* which had all been observed in the PSC group. No GO pathways reached multiple correction testing; top enrichment with nominal significance was found for myeloid leucocyte activation (*p* = 0.0001) and cell junction disassembly (*p* = 0.0004) (Supplementary Fig. 5).

**Table 1:** All 19 methylated sites found between IgG4-SC and healthy controls from EWAS

Rank	IlmnID	Gene	Chr	Location	BetaHealthy Control	Beta IgG4-SC	Beta diff	*P*. Val	Adj. *P*. Val
1	cg22914762	*MIR1973*	4	Body	0.226	0.292	0.066	1.10E-23	9.50E-18
2	cg12949141	*PCBD2*	5	Body	0.262	0.421	0.158	2.99E-23	2.59E-17
3	cg13170468		11		0.301	0.377	0.076	5.47E-16	4.74E-10
4	cg00490885	*MIR4485*	11	TSS1500	0.178	0.234	0.056	9.32E-14	8.07E-08
5	cg03964851	*MIR1974*	5	TSS200	0.775	0.739	− 0.036	5.42E-13	4.69E-07
6	cg23505966	*CEP97*	3	1stExon	0.043	0.035	− 0.008	7.90E-12	6.84E-06
7	cg05812299	*MTRNR2L5*	10	TSS200	0.139	0.174	0.035	1.00E-11	8.68E-06
8	cg16581032		8		0.137	0.164	0.027	1.78E-11	1.54E-05
9	cg26094004	*PYY*	17	5'UTR	0.414	0.486	0.071	3.15E-10	0.0003
10	cg23492258	*MTRNR2L5*	10	TSS1500	0.366	0.443	0.077	1.18E-09	0.0010
11	cg14069320	*IFNAR1*	21	TSS1500	0.046	0.036	− 0.010	1.63E-09	0.0014
12	cg23558456		1		0.663	0.757	0.094	2.19E-09	0.0019
13	cg27143246	*MYNN*	3	TSS1500	0.286	0.216	− 0.069	4.39E-09	0.0038
14	cg15077985		18		0.769	0.839	0.070	1.19E-08	0.0103
15	cg24866706		3		0.796	0.861	0.065	1.26E-08	0.0109
16	cg08422863	*HERC6*	4	Body	0.183	0.130	− 0.053	1.79E-08	0.0155
17	cg02279883	*TANC2*	17	TSS1500	0.882	0.863	− 0.019	2.51E-08	0.0217
18	cg16267121	*MTRNR2L3*	20	TSS1500	0.159	0.184	0.025	3.04E-08	0.0263
19	cg18840034	*WIBG*	12	Body	0.680	0.753	0.073	5.40E-08	0.0468

### Epigenome-wide association in PSC

**Fig. 2:**
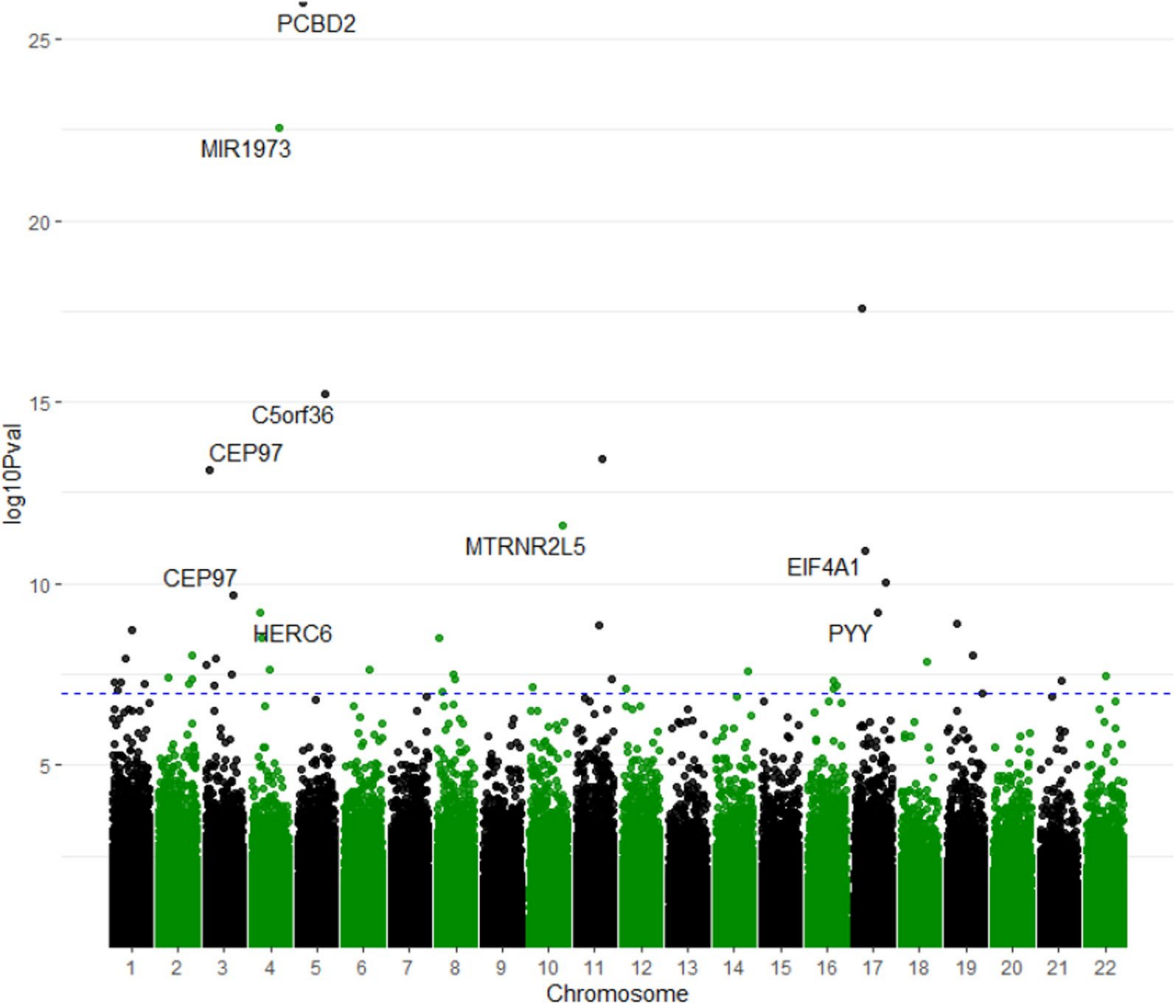
EWAS Manhattan plot showing differently methylated positions in patients with PSC when compared to healthy controls

Methylation analyses revealed that, when compared to control, patients with PSC had 38 differently methylated positions (DMP) after Holm correction (Table 2, Fig. 2, Supplementary Fig. 2A). Among these, *PCBD2* (*p* = 8.45 × 10^–21^), which is involved in tyrosine biosynthesis, micro-RNA 1973 (*p* = 2.45 × 10^–17^) and 1974/C5orf36 (*p* = 2.19 × 10^–12^), *CEP97* (*p* = 1.78 × 10^–4^), involved in calmodulin activity, and *MTRNR2L5* (*p* = 2.07 × 10^–6^), which regulates apoptosis, were the most differently methylated sites. Further analyses of differentially methylated regions (DMR’s) found a total of seven gene regions, including *MTRNR2L13, MTRNR2L8, MTRNR2L1, CEP97, TXK, CBFA2T3,* and *API5* (Supplementary Table 2). Gene ontology (GO) analysis showed enrichment for immune-mediated pathways including, T cell activation (*p* = 0.0004), lymphocyte activation (*p* = 0.0007), leucocyte differentiation (*p* = 0.0008) and activation (*p* = 0.0004) and mononuclear cell differentiation (*p* = 0.004) (Supplementary Fig. 3).

**Table 2:** All 38 methylated sites found between PSC and controls from EWAS

Rank	IlmnID	Gene	Chr	Location	Beta Healthy Control	Beta PSC	Beta diff	*P*. Val	Adj. *P*. Val
1	cg12949141	*PCBD2*	5	Body	0.2623	0.3845	0.1221	9.76E-27	8.45E-21
2	cg22914762	*MIR1973*	4	Body	0.2263	0.2760	0.0496	2.83E-23	2.45E-17
3	cg03992651		17		0.6044	0.6547	0.0503	2.54E-18	2.20E-12
4	cg03964851	*MIR1974*		TSS200	0.7745	0.7420	0.0325	6.07E-16	5.26E-10
5	cg13170468		11		0.3006	0.37019	0.06956	3.73E-14	3.23E-08
6	cg23505966	*CEP97*	3	1stExon	0.0431	0.0367	0.0063	7.76E-14	6.72E-08
7	cg23492258	*MTRNR2L5*	10	TSS1500	0.3664	0.4257	0.0592	2.40E-12	2.07E-06
8	cg07421515	*EIF4A1*	17	Body	0.5906	0.5699	0.0207	1.21E-11	1.05E-05
9	cg11212901		17		0.7479	0.7141	0.0338	9.30E-11	8.06E-05
10	cg24172986	*CEP97*	3	Body	0.0433	0.0380	0.0052	2.06E-10	0.0002
11	cg08422863	*HERC6*	4	Body	0.1825	0.1447	0.0378	6.04E-10	0.0005
12	cg26094004	*PYY*	17	5'UTR	0.4143	0.4708	0.0565	6.06E-10	0.0005
13	cg17667988	*PTBP1*	19	TSS200	0.0463	0.0388	0.0074	1.22E-09	0.0010
14	cg00490885	*MIR4485*	11	TSS1500	0.1778	0.2139	0.0361	1.35E-09	0.0012
15	cg23558456		1		0.6630	0.7207	0.0577	1.82E-09	0.0016
16	cg11132334	*TXK*	4	TSS200	0.3443	0.3811	0.0367	3.03E-09	0.0026
17	cg16581032		8		0.1373	0.1562	0.0189	3.12E-09	0.0027
18	cg20923498	*INPP4A*	2	5'UTR	0.5958	0.6265	0.0306	9.28E-09	0.0080
19	cg01774027	*ARID3A*	19	Body	0.3041	0.2723	0.0317	9.53E-09	0.0082
20	cg08345526	*ITPKB*	1	Body	0.6510	0.6939	0.0429	1.15E-08	0.0100
21	cg05044994	*LPP*	3	5'UTR	0.6484	0.6771	0.0287	1.20E-08	0.0104
22	cg27630863	*SMAD2*	18	5'UTR	0.2619	0.3057	0.0438	1.44E-08	0.0125
23	cg24866706		3		0.7959	0.8351	0.0391	1.63E-08	0.0141
24	cg01213536		6		0.5094	0.4944	0.0149	2.34E-08	0.0202
25	cg02600394	*TXK*	4	5'UTR	0.5747	0.6043	0.0295	2.36E-08	0.0204
26	cg06946814	*ZFYVE21*	14	Body	0.8487	0.8658	0.0171	2.58E-08	0.0223
27	cg15739904		8		0.4237	0.4699	0.0462	3.08E-08	0.0267
28	cg04044187		3		0.7676	0.7288	0.0387	3.12E-08	0.0271
29	cg10546888	*DDX17*	22	Body	0.3858	0.4276	0.0418	3.41E-08	0.0295
30	cg17509462	*BIN1*	2	Body	0.2501	0.2229	0.0272	3.86E-08	0.0334
31	cg06235847	*PLA2G16*	11	TSS200	0.0678	0.0588	0.0089	4.17E-08	0.0361
32	cg10639435	*ZNF250*	8	3'UTR	0.4087	0.4575	0.0488	4.27E-08	0.0369
33	cg12206840		2		0.5353	0.5745	0.0391	4.40E-08	0.0381
34	cg12281271	*ERG*	21	Body	0.4636	0.4898	0.0261	4.78E-08	0.0414
35	cg08614766		16		0.6947	0.7186	0.0239	4.93E-08	0.0426
36	cg00714585	*THEM5*	1	Body	0.2309	0.1879	0.0430	5.02E-08	0.0434
37	cg22519184	*LOC101928626*	1	TSS200	0.3676	0.3018	0.0658	5.38E-08	0.0466
38	cg00160981	*FCRLB*	1	TSS1500	0.2097	0.1836	0.0260	5.48E-08	0.0474

The comparisons of those with PSC only (n = 13) and PSC-UC (n = 57) revealed no differently methylated CpG sites after Holm correction (Supplementary Fig. 4).

### Overlapping methylation sites between IgG4-SC, PSC and UC

A total of five differently methylated probes were shared among PSC, IgG4-SC and UC (Table 3). Shared directionality of the probes was also observed between all diseases. IgG4-SC and PSC shared seven significant differences including multiple sites within *CEP97*, *MTRNR2L5*, *HERC6*, *MIR4485*, *LINC00293* and *EPHA6*. PSC and UC shared eight significant differences including two sites within *MTRNR2L1*, cg01213536, *EIF4A1*, *THEM5*, *PTBP1*, cg04044187 and *LOC101928626*. No overlap was found specific to IgG4-SC and UC that was not shared with PSC.

**Table 3:** Log fold change of overlapping sites found from individual EWAS’s between IgG4-SC, UC and PSC

IlmnID	Gene Name	Beta diff IgG4-SC	Adj. *P*. Val	Beta diff UC	Beta diff PSC	Adj. *P*. Val	Adj. *P*. Val
cg1294914	*PCBD2*	0.158	1.29 × 10^−17^	0.132	0.122	6.58 × 10^−24^	1.97 × 10^−15^
cg22914762	*MIR1973*	0.066	9.50 × 10^−18^	0.048	0.050	1.23 × 10^−17^	1.97 × 10^−15^
cg03964851	*MIR1974*	− 0.036	9.38 × 10^−8^	− 0.034	− 0.033	1.31 × 10^−10^	2.61 × 10^−11^
cg26094004	*PYY*	0.071	3.03 × 10^−5^	0.068	0.057	4.38 × 10^−5^	4.48 × 10^−9^
cg13170468		0.076	1.58 × 10^−10^	0.067	0.069	6.47 × 10^−9^	1.76 × 10^−7^

### Methylation Quantitative Trait Loci (meQTL) analysis

MeQTL analysis revealed 1891 methylation sites implicated by genetic SNPs for IgG4-SC and 4125 for PSC (Tables 4 and 5). Both cohorts showed clustering within the HLA region found on chromosome 6 with shared overlap at *HLA-DQB2*, *HLA-DPA1*, *HLA-F* and *HLA-DRA*.

**Table 4:** Top 20 meQTL’s observed in IgG4-SC

	snps	IlluminID	*P* Val	Adj. *P*. Val	Chr	Gene
1	rs76705950	cg03716678	9.77E-73	2.36E-66	8	*LOC101927588*
2	GSA-rs12310185	cg26941787	8.27E-70	9.97E-64	12	
3	rs6580395	cg09146088	1.05E-66	8.41E-61	5	
4	rs1671317	cg06500073	1.12E-65	6.78E-60	4	*DDX60*
5	GSA-rs10978942	cg06520293	1.74E-65	8.37E-60	9	
6	rs78957168	cg11276189	6.83E-62	2.74E-56	7	*LOC154822*
7	rs7708590	cg12471283	6.29E-61	2.17E-55	5	
8	rs1234612	cg26893861	8.82E-59	2.66E-53	17	*DUSP3*
9	rs3781648	cg01192554	2.39E-56	5.75E-51	11	*PPFIA1*
10	rs55877187	cg15625495	4.97E-52	1.09E-46	1	*VPS72*
11	rs10076268	cg15618908	1.41E-51	2.83E-46	5	
12	GSA-rs7037930	cg11078769	3.93E-47	7.29E-42	9	
13	rs1909116	cg24284460	4.49E-47	7.72E-42	3	*TRAK1*
14	GSA-rs17151639	cg07304760	5.86E-46	9.42E-41	7	*SND1*
15	rs2074872	cg13984289	9.29E-45	1.40E-39	17	*MYH13*
16	GSA-rs1251079	cg10523679	3.06E-44	4.10E-39	1	*ACADM*
17	rs72810983	cg18693985	4.48E-44	5.40E-39	5	*CPEB4*
18	rs3827900	cg27560391	1.23E-43	1.36E-38	14	*DDX24*
19	rs4141377	cg18446441	1.24E-43	1.36E-38	13	*EEF1DP3*
20	rs2741689	cg08060988	8.64E-43	9.05E-38	8	*DEFA6*

**Table 5:** Top 20 meQTL’s observed in PSC

	SNPs	IlluminaID	*P*. Val	Adj. *P*. Val	Chr	Gene
1	rs375984	cg26021304	9.48E-82	7.86E-77	6	*ZFP57*
2	rs9268839	cg11294950	9.57E-82	7.86E-77	6	
3	GSA-rs7193473	cg05477582	1.08E-80	7.35E-76	16	*CMTM1*
4	rs1883847	cg09864671	1.41E-80	8.25E-76	20	*DIDO1*
5	rs8019916	cg23022053	1.48E-78	7.59E-74	14	*PTGDR*
6	rs4148943	cg05125667	2.93E-77	1.34E-72	10	*CHST3*
7	GSA-rs36178	cg07068406	4.37E-77	1.79E-72	3	*EPHB1*
8	GSA-rs11175307	cg11601920	3.74E-76	1.40E-71	12	*C12orf56*
9	rs1997243	cg03916490	9.93E-74	2.16E-69	7	*C7orf50*
10	rs9268127	cg13966843	1.21E-73	2.16E-69	6	*C6orf10*
11	rs3117582	cg23634079	1.69E-72	1.98E-68	6	*MSH5*
12	rs798544	cg24648384	3.54E-72	4.04E-68	7	*GNA12*
13	rs1997243	cg22785556	2.41E-71	2.67E-67	7	*C7orf50*
14	rs9977496	cg16536985	5.24E-70	5.20E-66	21	
15	rs1023449	cg07389699	5.45E-70	5.20E-66	6	*HLA-DQB2*
16	rs2015845	cg17406915	1.47E-68	1.31E-64	19	
17	rs3095152	cg04559908	2.45E-68	2.05E-64	6	*DPCR1*
18	rs2523607	cg22731440	2.24E-66	1.74E-62	6	*HLA-B*
19	rs929157	cg05074385	5.10E-66	3.37E-62	6	*TRIM15*
20	rs9268839	cg13081526	3.32E-65	2.06E-61	6	

### Epigenetic clock

**Fig. 3:**
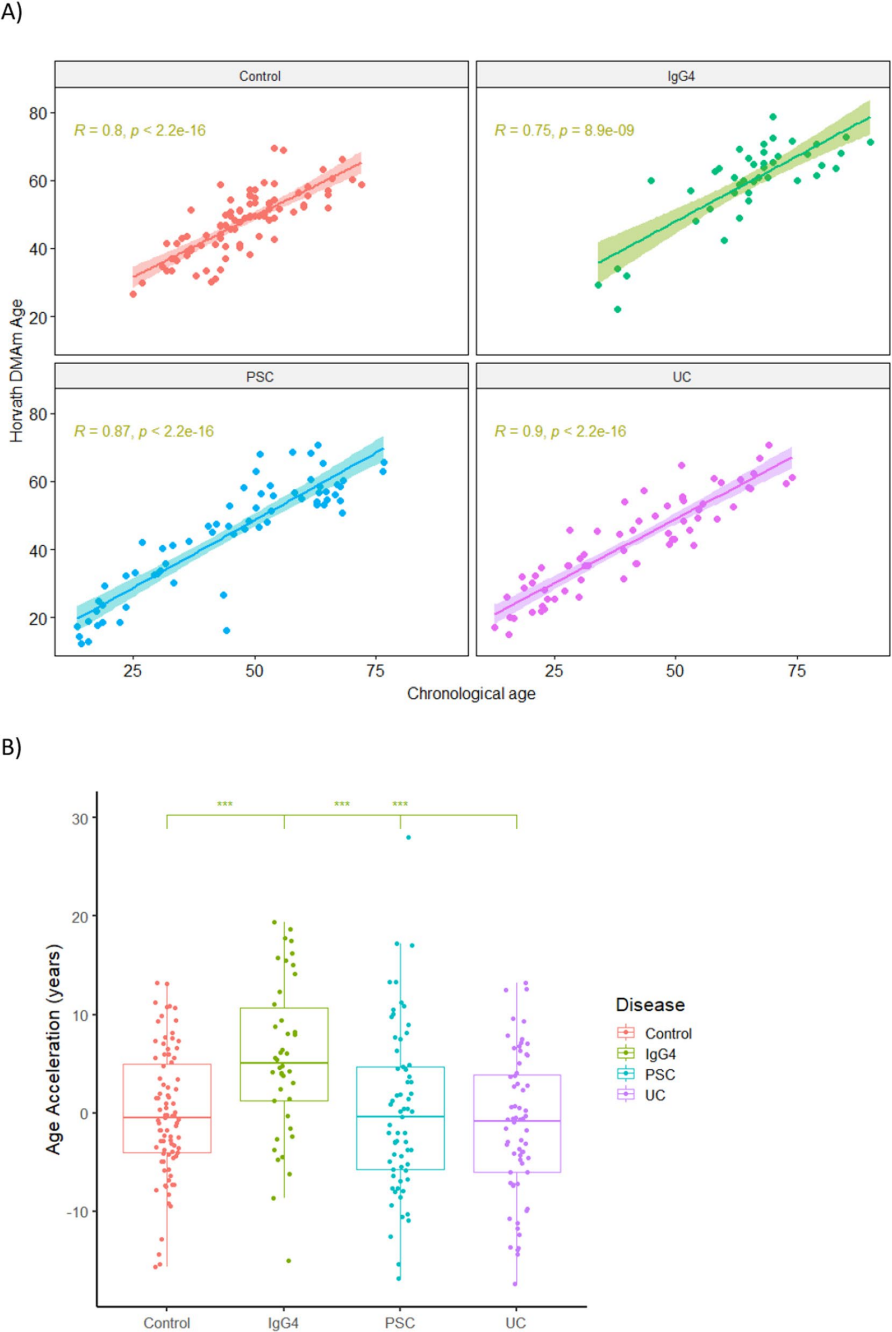
Epigenetic ageing estimated using Horvath’s Clock. **A** Correlation between expected epigenetic age and chronological age; **B** age acceleration distribution in each group (bars represent SD and bold lines inside the box plot median levels). Levels of significance: ***p = 0.001; using Kruskal–Wallis test

Expected epigenetic calculated using the Horvath’s clock was strongly correlated with chronological ages for PSC, IgG4-SC, UC and controls (Fig. 3A). Significant age acceleration was observed between IgG4-SC cohort and controls (*p* = 0.0001) (Fig. 3B). No significant correlation was found between increased epigenetic age and active disease in IgG4-SC patients compared to those who were in remission at the time the blood was sampled (*p* = 0.876). No age acceleration was found between either PSC or UC compared to controls.

## Discussion

This is the first study to provide a comprehensive comparative analysis of the methylome in patients with IgG4-SC and PSC. We identify specific methylation changes in both disease groups compared with controls, with 19 and 38 significant CpGs found in IgG4-SC and PSC, respectively. A proportion of sites were shared between both diseases and with UC, including *PCBD2*, *PYY*, microRNAs miR-1973 and miR-1974. Furthermore, IgG4-SC and PSC also shared multiple differentiated sites independently of UC, including *CEP97*, *MTRNR2L5*, HERC6, *MIR4485*, *LINC00293* and *EPHA6*. Our findings represent new insights into pathophysiology mechanisms as well as similarities between these two conditions.

We also uncovered a strong interplay between genetic variation and DNA methylation in both IgG4-SC and PSC, particularly at the HLA locus, which is central to immune function. Several HLA genes are known to be implicated in PSC susceptibility, although the mechanisms involved have remained uncertain. Here, we found that *HLA-DPA1* and *HLA-DQA1*, *HLA-DRB1* represent meQTL loci in PSC and the later in IgG4-SC underscoring potential explanation that DNA methylation may mediate the HLA association in PSC as well as in other immune-mediated diseases. Of further relevance is the observation that *HLA-DQA2* undergoes epigenetic modulation in CD4 + T cells of patients with IgG4-RD [39].

Our findings corroborate previous reports that modulation of gene expression through epigenetic changes is present in PSC. For instance, *ETS1* and *CDKN2A* are involved in resistance to apoptosis in senescent cholangiocytes of patients with PSC, where chromatin remodelling and *ETS1* serve as transcriptional regulators of *CDKN2A* [40]. This promotes production of pro-inflammatory cytokines by senescent cholangiocytes and the perpetuation of biliary injury in patients. In our cohort, *ETS1* was also identified as a meQTL in PSC thus reinforcing the role of genetic variation and its link to differential DNA methylation. Several other genes previously associated with PSC were also identified in our meQTL analysis, such as *UBASH3A, IL2RA, CD226, CCDC88B, SOCS1, MAX, GNAS, PTPRN2* and *IRF5* [12, 41, 42]. Recently, *ETS2* was highlighted as a potential driver of inflammation among several immune-mediated diseases, including IBD and PSC [43]. *ETS2* functions as a regulator of macrophages and monocytes, creating a microenvironment that favours inflammation. In our analysis, *ETS2* was identified as a meQTL in IgG4-SC. This brings into question whether macrophages play a role in IgG4-RD pathogenesis. Other risk loci described in immune-mediated diseases have also been identified as meQTLs in IgG4-SC, namely *GPR35, PTPRN2, CLEC16A, HKR1, VENTX, STK11, SLC12A7* and *MBP* [12, 39, 42]. The commonalities found in these analyses between PSC and IgG4-SC suggest that these diseases may share mechanistic pathways and that understanding the pathogenesis of one might inform us about the other as well.

Epigenetic age acceleration has been described in patients with PSC, and it has been associated with worse clinical outcomes [44]. Age acceleration represents the difference between biological age and chronological age [37]. This measure of molecular ageing has been associated with several age-related diseases, including progression of disease in IBD, increased risk of cardiovascular, cancer, and all-cause mortality [45–48]. Using the Horvath’s clock, we observed age acceleration in IgG4-SC compared to PSC, UC, and control groups despite more than 50% of the IgG4-SC patients being in remission. We hypothesise that the chronic and subclinical inflammation observed in patients through advanced epigenetic ageing contributes to the substantial risk of cardiovascular disease and cancer in this population, namely 69-fold increase in the risk of lymphoma and fourfold increase for pancreatic cancer [49].

Interestingly, our analysis did not identify age acceleration in patients with PSC, contrary to previous reports, including the phase IIb clinical trial of simtuzumab [44]. A high degree of age acceleration correlated with a poorer clinical outcome (i.e. ascending cholangitis, hepatic decompensation, liver transplantation, cholangiocarcinoma) in these patients. Differences in patient populations such as the more advanced staged cohort (64% Ishak F5-6) with multiple samples over time, and the methodological variations, including the post-hoc control group may explain the discrepancies observed. The resolution of age acceleration with treatment of the underlying condition is variable, and likely depends on differences between conditions, the timescales, and the tissues studied.

## Conclusion

This study offers novel insights into the epigenetic landscape of IgG4-SC and PSC, highlighting the significant overlap in methylome profiles between the diseases. The interplay between germline variants and DNA methylation in these diseases, particularly in the HLA region, underscores the critical role of the immune system in the pathogenesis of IgG4-SC and PSC. Furthermore, patients with IgG4-SC show epigenetic age acceleration despite disease remission, which might imply chronic inflammation and explain the increased risk of cardiovascular diseases and cancer in this population.

## Supplementary Information

Additional file 1.

## Data Availability

Normalised data can be available on reasonable request.

## Abbreviations

IgG4-SC: IgG4-related cholangitis
PSC: primary sclerosing cholangitis
UC: ulcerative colitis
HC: healthy controls
HLA: human leukocyte antigens
IBD: inflammatory bowel disease
DMP: differentially methylated position
FDR: false discovery rate
DMRs: differentially methylated regions
GO: gene ontology
HISORt: histology imaging serology other organ involvement and response to therapy
IgG4-RI: IgG4-responder index
EASL: European association for the study of the liver
SNPs: single nucleotide polymorphisms
