# Supplementary material for: Epigenetic age acceleration and methylation differences in IgG4-related cholangitis and primary sclerosing cholangitis
Source: Clin Epigenetics. 2025 Jan 16;17:6. doi: 10.1186/s13148-024-01803-x (PMC11740490; doi:10.1186/s13148-024-01803-x)
Supplement: Supplementary file 1 — Additional file 1. [file 13148_2024_1803_MOESM1_ESM.docx]

Supplementary Figures:

Supplementary Figure 1: Estimated cell type proportions observed within each of the disease groups.


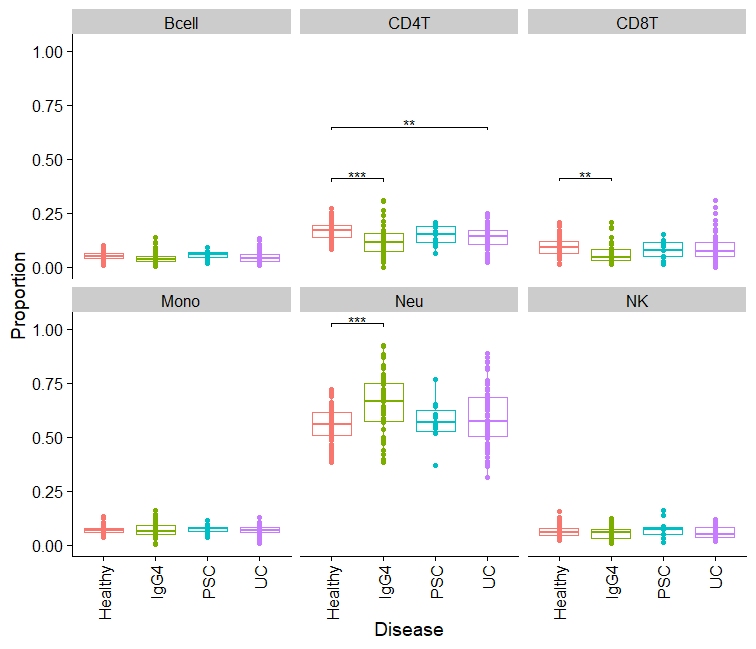


Supplementary Figure 2: Measured genomic inflation using Q-Q plots

A)
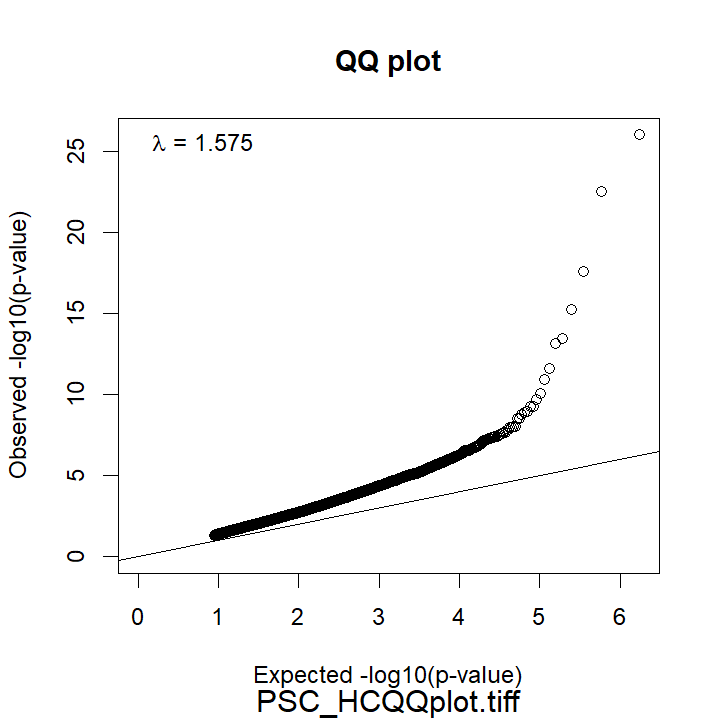


B)


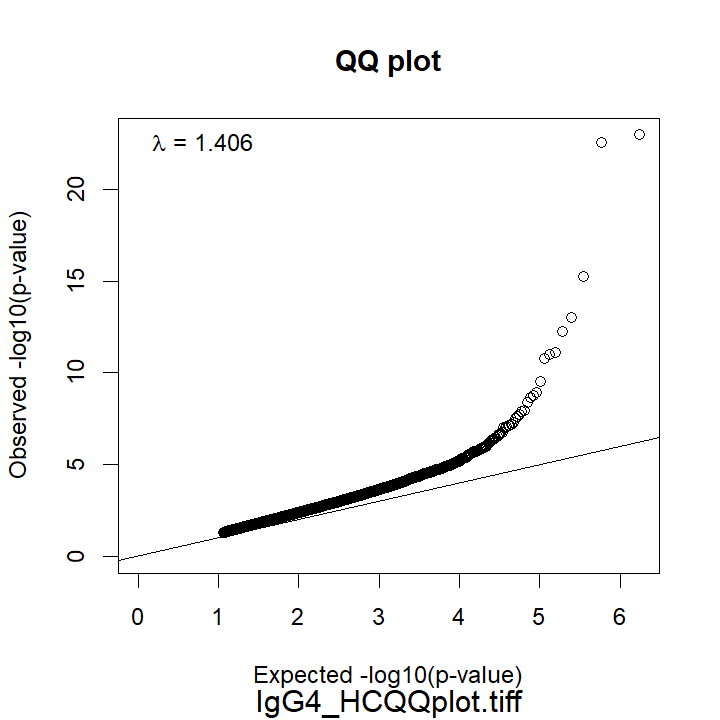


Supplementary Figure 3: Gene ontology enrichment for PSC


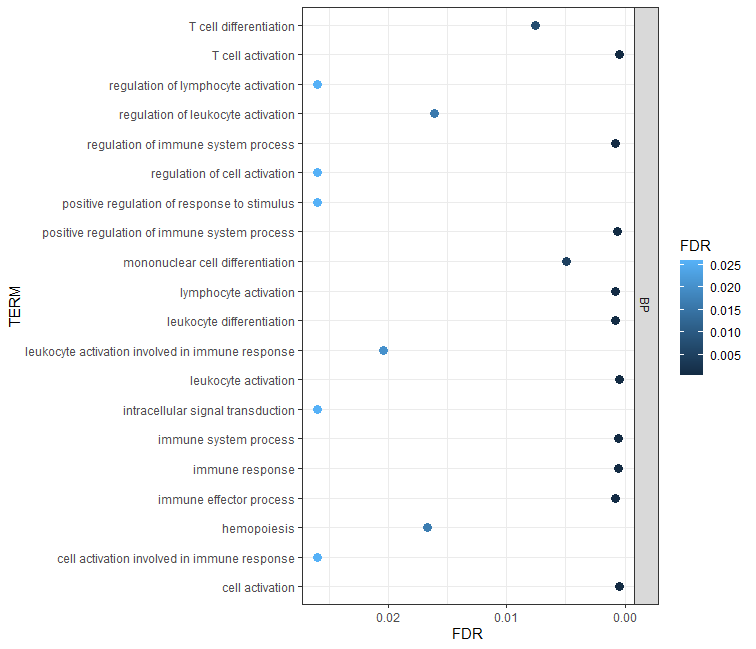


Supplementary Figure 4: Manhattan plot of the EWAS findings between PSC only and those with PSC-UC.


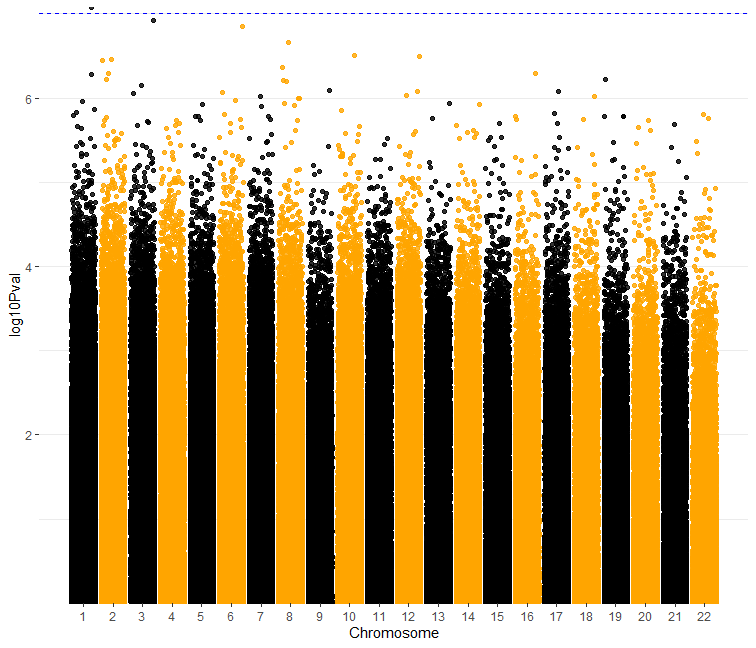


Supplementary Figure 5: Gene ontology analysis of IgG4-C.


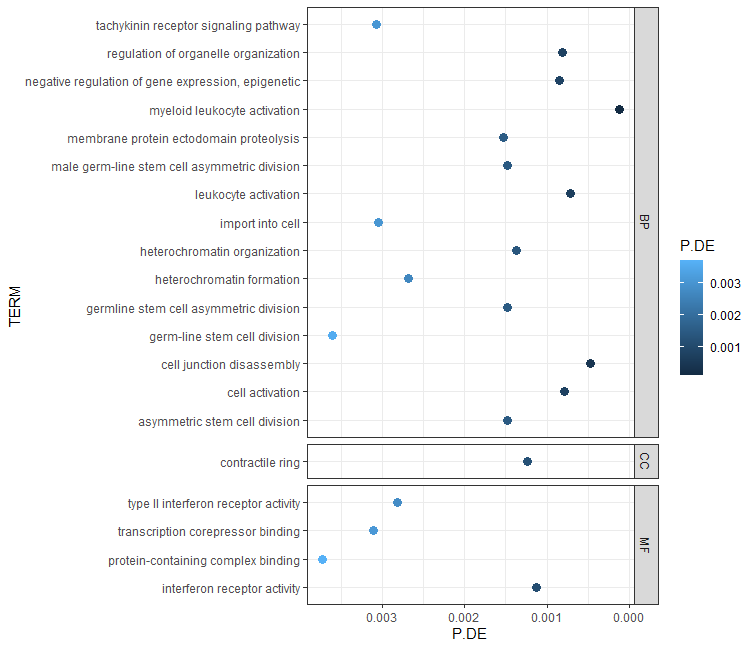


Supplementary Table 1: Demographic and clinical information

|  | IgG4-SC (n=47) | PSC (n=65) | UC (n=64) |
| --- | --- | --- | --- |
| Median age at diagnosis (IQR) | 63 (12) | 46 (36) | 24 (15) |
| Male | 38 (81%) | 44 (68%) | 31 (48%) |
| Median disease duration (IQR) | 2 (3) | 6 (8) | 10 (18) |
| Immunosuppressive medication | 14 (30%) | 17 (26%) | 27 (42%) |
| Smoking status |  |  |  |
| Smoker | 3 | 1 | 2 |
| Ex-smoker | 15 | 1 | 10 |
| Never smoked | 25 | 50 | 18 |
| Unknown | 3 | 12 | 31 |
